# Supplementary figures and images for: Intra- and inter-operator reliability of measuring compressive stiffness of the patellar tendon in volleyball players using a handheld digital palpation device
Source: PLoS One. 2024 Jun 25;19(6):e0304743. doi: 10.1371/journal.pone.0304743 (PMC11198853; doi:10.1371/journal.pone.0304743)

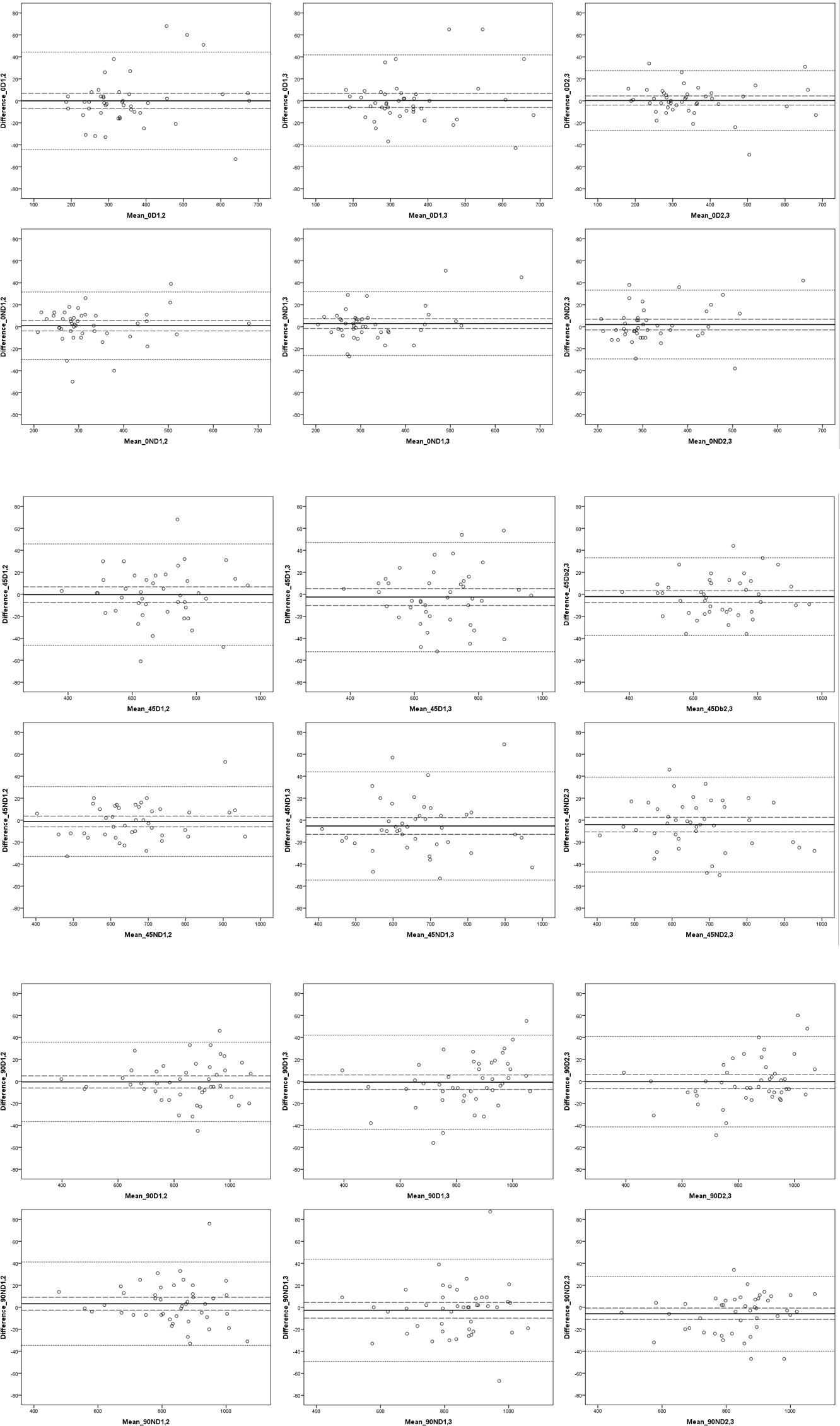

Supplement: S1 Fig — Upper row: dominant knee, lower row: non-dominant knee. Since measurements were performed in triplicate and with Bland-Altman plots only two can be compared; the columns from left to right represent the comparisons between measurements 1 and 2, measurements 1 and 3, and measurements 2 and 3. In each figure, the bold black line represents the mean of the difference, the small dotted lines represent the upper and lower limits of agreement (LOA), and the long dotted lines the 95% confidence interval of the mean difference. B.1: 0° knee flexion, B.2: 45° knee flexion, B.3: 9° knee flexion. (TIF) [file pone.0304743.s001.tif]
