# Supplementary material for: Intra- and inter-operator reliability of measuring compressive stiffness of the patellar tendon in volleyball players using a handheld digital palpation device
Source: PLoS One. 2024 Jun 25;19(6):e0304743. doi: 10.1371/journal.pone.0304743 (PMC11198853; doi:10.1371/journal.pone.0304743)
Supplement: S1 Table — (DOCX) [file pone.0304743.s002.docx]

**Table S1. Compressive stiffness.**

| Knee | Angle | Male (n=29) | Female (n=16) | p-value |
| --- | --- | --- | --- | --- |
| Dominant | 0º | 386.6±130.5 | 287.8±77.8 | 0.003 |
|  | 45º | 714.0±128.6 | 609.0±87.9 | 0.004 |
|  | 90º | 883.8±133.7 | 745.4±151.1 | 0.005 |
|  | p-value | <0.001 | <0.001 |  |
| Non-dominant | 0º | 344.2±105.9 | 298.1±54.1 | 0.060 |
|  | 45º | 687.6±135.8 | 615.0±77.3 | 0.035 |
|  | 90º | 871.4±110.2 | 764.2±124.4 | 0.008 |
|  | p-value | <0.001 | <0.001 |  |

P-values shown represent the differences between knee angles and between sexes.
