# Supplementary material for: Intra- and inter-operator reliability of measuring compressive stiffness of the patellar tendon in volleyball players using a handheld digital palpation device
Source: PLoS One. 2024 Jun 25;19(6):e0304743. doi: 10.1371/journal.pone.0304743 (PMC11198853; doi:10.1371/journal.pone.0304743)
Supplement: S2 Table — (DOCX) [file pone.0304743.s003.docx]

**Table S3. P-values of the differences in reliability.**

| Knee | Angle | P-value differences in reliability between sexes | P-value differences in reliability over age* |
| --- | --- | --- | --- |
| Dominant | 0º | 0.318 | r=-0.093, p=0.541 |
|  | 45º | 0.800 | r=-0.021, p=0.892 |
|  | 90º | 0.237 | r=-0.181, p=0.235 |
| Non-dominant | 0º | 0.271 | r=0.251, p=0.097 |
|  | 45º | 0.498 | r=0.031, p=0.838 |
|  | 90º | 0.941 | r=0.033, p=0.828 |

 *Pearson correlation value included.
